# Photonic-dispersion neural networks for inverse scattering problems

**DOI:** 10.1038/s41377-021-00600-y

**Published:** 2021-07-27

**Authors:** Tongyu Li, Ang Chen, Lingjie Fan, Minjia Zheng, Jiajun Wang, Guopeng Lu, Maoxiong Zhao, Xinbin Cheng, Wei Li, Xiaohan Liu, Haiwei Yin, Lei Shi, Jian Zi

**Affiliations:** 1 State Key Laboratory of Surface Physics, Key Laboratory of Micro- and Nano-Photonics Structures (Ministry of Education) and Department of Physics, Fudan University, Shanghai 200433, China; 2 Shanghai Engineering Research Center of Optical Metrology for Nano-fabrication (SERCOM), Shanghai 200433, China; 3 Institute of Precision Optical Engineering, School of Physics Science and Engineering, Tongji University, Shanghai 200092, China; 4 National Institute of Metrology, Beijing 100029, China; 5 Collaborative Innovation Center of Advanced Microstructures, Nanjing University, Nanjing 210093, China

**Keywords:** Near-infrared spectroscopy, Photonic crystals

## Abstract

Inferring the properties of a scattering objective by analyzing the optical far-field responses within the framework of inverse problems is of great practical significance. However, it still faces major challenges when the parameter range is growing and involves inevitable experimental noises. Here, we propose a solving strategy containing robust neural-networks-based algorithms and informative photonic dispersions to overcome such challenges for a sort of inverse scattering problem—reconstructing grating profiles. Using two typical neural networks, forward-mapping type and inverse-mapping type, we reconstruct grating profiles whose geometric features span hundreds of nanometers with nanometric sensitivity and several seconds of time consumption. A forward-mapping neural network with a parameters-to-point architecture especially stands out in generating analytical photonic dispersions accurately, featured by sharp Fano-shaped spectra. Meanwhile, to implement the strategy experimentally, a Fourier-optics-based angle-resolved imaging spectroscopy with an all-fixed light path is developed to measure the dispersions by a single shot, acquiring adequate information. Our forward-mapping algorithm can enable real-time comparisons between robust predictions and experimental data with actual noises, showing an excellent linear correlation (*R*^2^ > 0.982) with the measurements of atomic force microscopy. Our work provides a new strategy for reconstructing grating profiles in inverse scattering problems.

## Introduction

Inverse scattering problems (ISPs) arise in many fields of science and engineering such as computed tomography^1,2^, fiber Bragg gratings^3^, and optical metrology^4–7^. A typical ISP, is composed of three parts: a set of scattering objectives, a set of light responses and a measurement operator. For scattering objectives, one should make a parameter space whose elements are arrays of parameters, describing the scatters’ geometries and components; for light responses, a data space is needed whose elements correspond to the measured optical responses of scatters in the far field, such as reflectance spectra. As the connection of these two sets, a measurement operator characterizes the mapping from parameter space to data space. To solve ISPs, namely inferring an element of the parameter space from that of the data space, it executes the inversion of the measurement operator—inversion operator. Two key properties of the inversion operator are its injectivity and stability^8^. Injectivity requires the acquired data to uniquely characterize the parameters, and stability is closely related to the measurement noises.

Many algorithms and measuring techniques have been developed to solve ISPs with good injectivity and stability. In terms of algorithms, the genetic algorithm^9^ and library approach^10^ stand out with their understandability and feasibility. However, the existing algorithms are usually time-consuming due to the global optimization of a huge parameter space. Recently, neural networks^11,12^ (NNs) have offered a new perspective to solve inverse problems^13–17^; for instance, by inverse structure design^18–27^. But when applied to practical ISPs, the performance of NN-based algorithms suffers from inevitable measuring noises, showing low stability. As for measuring techniques, high-throughput measuring methodologies such as Mueller matrix ellipsometry^28^ are of great practical importance to provide adequate information for mapping algorithms. Redundant information ensures the injectivity of mapping, but it brings in sort of extra vibration instability when detecting multi-dimensional signals by mechanical modules. Thus, it is still a challenge to perform a rapid stable high-throughput measurement by a single-shot imaging technique.

To obtain a technique with both injectivity and stability, we develop a high-throughput Fourier-optics-based angle-resolved imaging spectroscopy (ARS) embedded with robust NN-based algorithms to solve ISPs. Our solving strategies are experimentally applied to a particular ISP of reconstructing the silicon-on-insulator (SOI) grating profiles with nanometric-scale precision. We discuss two kinds of NN-based algorithms: One is the inverse-mapping algorithm, and the other is the forward-mapping-based optimization algorithm—forward-mapping algorithm. We first train an inverse-mapping NN to learn the inversion operator, directly mapping from data space to parameter space. On the other side, the forward-mapping NN is trained to learn the measurement operator from parameter space to data space, and a gradient-based optimization is further performed on the parameter space to find the optimal solution. Both algorithms are able to reconstruct the SOI grating profiles in the parameter space, whose size is orders of magnitude larger than those of the traditional methods (whose covered ranges of considered parameters are usually no more than 20 nm). The consumed mapping time is at the level of seconds, in which the inverse mapping costs < 1 s while forward mapping costs around 20 s. Considering the performance on experimental data, the forward-mapping algorithm shows more robustness to actual measurements and enables a real-time comparison of the responses after the solving process. In addition, we propose that the dispersions of a scattering objective can be used as the elements of data space. The structure information is contained in the shapes of dispersion curves labeled by wavelength (*λ*) and angle (*θ*), besides the absolute quantity of the reflection intensity, offering multi-dimensional information. Using the home-made ARS, we experimentally obtain the dispersion patterns with the all-fixed light path by single shot imaging. When armed with the NN-based algorithms, the reconstructed geometric parameters achieve a strong linear correlation (*R*^2^ > 0.982) with the measurements of atomic force microscopy (AFM).

## Results

In this section, we mainly discuss the key technical innovations in both mapping algorithms and measuring methodology, and our feasible strategy is further performed to reconstruct the SOI grating profiles from detected dispersions. For the algorithms, we focus more specifically on the details of the method using the forward-mapping algorithm in the main text, and those for the inverse-mapping algorithm are given in Supplementary Information.

### Overview of the algorithms

**Fig. 1:**
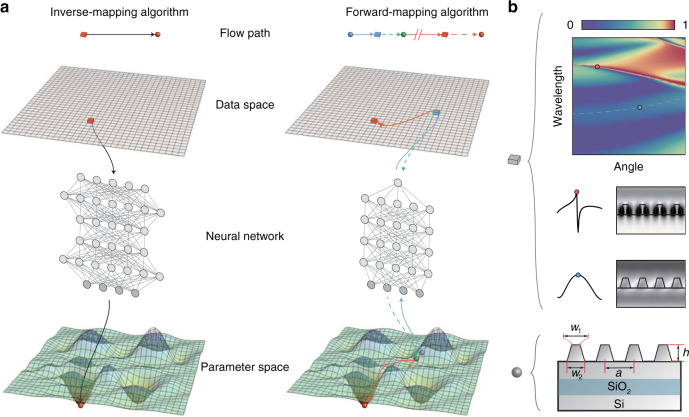
Two NN-based algorithms for solving ISPs. **a** Schematic overview of the solving process of two NN-based algorithms. The top space is the data space; the bottom space is the parameter space. The arrows are flow paths to describe the steps of algorithms. The fluctuations in parameter space stand for the difference between the detected response and theoretical response at a local point. **b** Upper panel: Each representative point (block) in the data space stands for a theoretical dispersion corresponding to the parameter space. The corresponding electric field distributions of the points in the dispersion are shown together with their angle-resolved spectra. Lower panel: Each representative point (ball) in the parameter space stands for a group of geometric parameters and components. The grating structure is modeled using four parameters: top line width *w*_1_, bottom line width *w*_2_, pitch *a* and height *h*

For ISPs, the discussions are usually expanded between the parameter space and the data space, as illustrated in Fig. 1a. Each representative point (ball) in the parameter space stands for a group of geometric parameters and components; each representative point (block) in the data space stands for the detected responses corresponding to a ball in the parameter space. The aim of solving ISPs is to try to establish an inverse mapping from data space to parameter space, inferring the parameters of the scattering objectives from the given detected (light) responses. Since the core of the inverse problems is to characterize the inversion of the measurement operator, it is natural to train a NN as a recognizer performing the inverse mapping directly, called inverse-mapping algorithm as shown in the left panel of Fig. 1a. Once the NN recognizer is trained as an inverse operator, the inverse mapping can solve ISPs in a straightforward way. The solving process is quite intuitive for the inverse-mapping algorithm: a detected response of the scattering objective (red block) enters the inverse-mapping NN that is previously trained on the simulated data sets, and the prediction of the parameters is further output by NN (red ball). To practically train an inverse-mapping NN is a challenge, because in most ISP cases the objective response can be viewed as the theoretical response superimposed with a measurement noise. Although the noise compared with signals is weak, it could be amplified by the inverse-mapping NN, leading to enormous deviations of predictions.

To show such noise influence, we start with an inverse-mapping NN trained on the simulated examples without noises. On a noise-free test set, 98.7% predicted parameters had deviations of < 1 nm after 400 epochs of training. However, the performance on the test set with Gaussian noises (*μ* = 0, *σ* = 0.1) became unsatisfactory (Fig. S1). A direct method to overcome that is to augment the data set during the training period, practically by adding some types of random noises, including Gaussian noise, to the theoretical responses. At this time, performance on the same noisy test set was improved greatly such that 97.8% predicted parameters had deviations of < 5 nm. It means that the robustness of inverse-mapping NNs can be enhanced by data set augmentation with the corresponding type of noise. But unfortunately, some unexpected noises in measurements still lead to unstable results. In this regard, due to the intrinsic architecture of the inverse-mapping NN, it has no ability to further tune the predicted parameters subtly.

For another way, the NN can be trained as a generator to generate responses, called the forward-mapping algorithm, as shown in the right panel of Fig. 1a. Once the NN generator is trained as a substitute for simulation algorithms, the whole architecture of the forward mapping can be entirely analytical, enabling us to calculate the gradients of the input parameters directly with the back propagation algorithm. Specifically, the optimization process starts from a random point (blue ball) in the parameter space. Entering the NN generator along the blue arrow, the array of random initial parameters is mapped to the corresponding response (blue block). The difference between the generated response R_g_ and the detected response R_d_ (red block) is described with a cost function, for instance, mean square error *C* = ∑|*R*_d_ - *R*_g_|^2^, reflecting in the fluctuations in parameter space. The gradient can therefore be defined as $\nabla _{\mathbf{p}}C$, where P stands for the parameters, as depicted by the green dash line in Fig. 1a. With the calculated gradient, the parameters (green ball) can be updated along the red arrow using some advanced gradient descent algorithms. After repeating the above steps for a few times, the optimization process will finally find the optimal point (red ball) in the parameter space. To prevent converging to a locally optimal solution, the optimization process described above usually starts simultaneously from several initial points in the parameter space, and the candidate solution is picked out with the smallest *C*. So far, the gradient descent algorithm plays to its rapid convergence ability to approach the globally optimal solution. Gradient-free algorithms, such as the search algorithm or greedy algorithm, are finally performed to search for the final solution starting with the selected candidate solution. Considering the actual response with measurement noises, the gradient-free algorithm enables the forward-mapping algorithm to finely tune the parameters in parameter space to find a solution whose corresponding response is nearest to the measured one. The detailed results of the forward-mapping algorithm applied to ISPs are given in the following sections.

### Data space element: photonic dispersion

Many kinds of light responses can be chosen as the elements of the data space. As an instance, Mueller matrix is usually used to describe the modulations of polarization states of an objective. Though almost all of the structure information is contained in the elements of the Mueller matrix, yet it needs to meet tough conditions such as stable rolling cantilevers and superhigh signal-to-noise ratio. Here, we experimentally propose a new kind of light response for solving ISPs: the photonic dispersions of the grating, characterized by the wavelength–angle (*λ–θ*) mapping. A tremendous number of researches in nanophotonics have revealed that wealthy accessible information lies in photonic dispersions^29–32^ such as photonic band structures and iso-frequency contours. Solving inverse problems in photonic crystals with photonic band structures has been reported by recent works: Wei et al.^16^ established an inverse-mapping algorithm with a convolutional NN to predict the Zak phase of 1D photonic crystals precisely from input Hamiltonians. Christensen et al.^33^ trained a convolutional NN and generative adversarial networks to predict and design inverse photonic crystal band structures with orders of-magnitude speedup. In our case, a measured photonic dispersion that contains both abundant band structures features and reflectance information is used to solve ISPs. A typical dispersion of an SOI grating with s-polarized light excitation is shown in Fig. 1b. The dispersion bands depicted by the observed stripes stem from different physical mechanisms. For example, one broad dispersion band, marked as a blue dashed curve, can be interpreted as the thin-film interference, while one Fano-shaped dispersion band, marked as a red dashed curve, is caused by the coupling between guided resonances and thin-film oscillations^34,35^. The various kinds of dispersive information can be further understood by their corresponding field distributions: Fields represented by the red point are extremely enhanced at the gratings, while those represented by the blue point are almost evenly distributed in the space. In this way, the grating structures can somehow injectively be in accordance with the dispersions labeled by (*λ*, *θ*), since the labeled detected intensities as well as the stripe-formed shapes could be regarded as the ruler measuring how strongly detected lights can interact with the grating structures. Thus, in this work, we use photonic dispersions as our data space elements. Besides, the grating profile can be modeled as isosceles trapezoids with four parameters: top line width *w*_1_, bottom line width *w*_2_, pitch *a* and height *h*. These geometric parameters vary in a range of hundreds of nanometers, constituting a parameter space of huge sizes. Specifically, we consider the line widths between 130 nm and 330 nm with the bottom line width longer than the top, pitch between 350 nm and 550 nm, and height between 160 nm and 270 nm.

### Forward-mapping NN

We observe sharp features and concretely abrupt changes at several wavelengths on the reflectance spectra, which are quite general in scattering problems. Then the first and foremost step in the forward-mapping algorithm is to train a NN that enables to generate such sharp features with high degrees of precision. Using a NN to generate high-quality factor resonance has essential implications, since it is one of the crucial properties in nanostructure and has attracted attention to the enhancement of light–matter interactions^36^. We firstly train a NN with the typical parameters-to-spectrum architecture, namely forward mapping the input geometric parameters to the whole spectrum in one time, to generate the dispersions. However, because of the correlations between two neighboring neurons in the output layer, we find such a NN that can only generate thin-film-interference features but fail in sharp Fano-shaped ones in a large–wavelength region (Fig. S3).

**Fig. 2:**
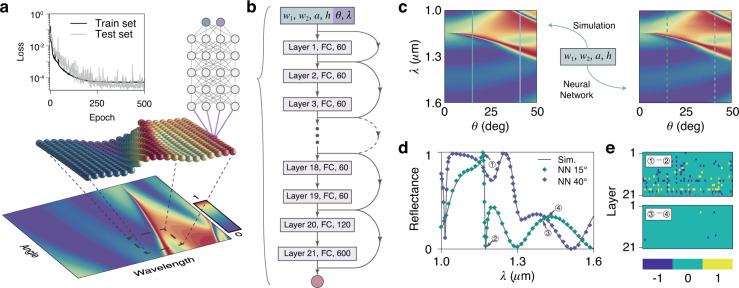
Architecture and performance of the forward-mapping NN. **a** Generation process and its training loss (inset). **b** Parameters-to-point architecture of the forward-mapping NN. **c** Comparison between the simulated (left) and NN-generated (right) dispersions of the SOI grating. Geometric parameters of SOI grating: *w*_1_ = 215 nm, *w*_2_ = 235 nm, *a* = 500 nm, *h* = 256 nm. Here, we just show the generated dispersion with s-polarized incident light, and that with p-polarization is shown in Fig. S4. **d** The simulated (black lines) and NN-generated (squares) dispersions are sliced at 15° and 40° for detailed comparisons. We lowered the sampling frequency of the generated dispersions for a better demonstration. Both of the sliced spectra contain at least one Fano-shaped feature. **e** Changes in the states of activation of neurons when NN generates two neighboring points of the reflectance in the dispersions

To overcome this deficiency, we develop a parameters-to-point forward-mapping NN with a different generating process, as illustrated in Fig. 2b. The word point here stands for a pixel (the ball connected with NN in Fig. 2a) on a dispersion pattern (reflectance at a single certain wavelength *λ* and a single certain angle *θ*). Besides the grating parameters, the NN inputs also include the labeled coordinates (*λ*, *θ*) of the dispersions and the output gives the corresponding reflectance. Such NN is realized by a residual fully-connected network with 21 layers: 60 neurons per layer in the former 19 layers, and 120 neurons and 600 neurons in the last two layers. Curving arrows between layers stand for the shortcuts of the residual blocks^37^. Batch normalization is applied before each nonlinear layer^38^. We use a rigorous coupled-wave analysis^39^ (RCWA) method to simulate the reflectance of 5 × 10^7^ points to set up a training set (see the Supplementary Information). Labels (*w*_1_, *w*_2_, *a*, *h*, *λ*, *θ*) of these samples form a six-dimensional space, where the reflectance data can be sampled with the Monte Carlo method. We have then trained the NN with an Adam optimizer^40^ on the training set, with the training error plotted in the inset of Fig. 2a. After training, the generation process of a complete dispersion pattern is realized by the pixel-by-pixel strategy: varying generating point coordinates (*λ*, *θ*) making NN scan on the generation region to calculate the reflectance of each pixel. (In practice, the scanning process is performed in parallel.) With desired resolutions, the intervals of (*λ*, *θ*) can be tuned finely.

To verify the performance of our proposed NN, we did a comparison between the simulated and NN-generated dispersions (Fig. 2c). The slices of two dispersions are further compared in Fig. 2d, showing a high accuracy of the NN generation capability. It also should be pointed out that our NN does indeed perform well in generating sharp spectra with Fano-shaped features. For instance, when moving from point 1 towards point 2 within 10 nm along the green spectrum, the reflectance has an abrupt fall with a change of almost one. To explore why the new NN was able to generate such sharp features, we checked the changes in activation states of the neurons. We use three colors to present the state changes [Fig. 2e]: blue stands for neurons switching from active states to inactive states, yellow for an inverse way, and green for those keeping the same states. We chose four points on the green spectrum for interpretation: Each of the groups (1,2) and (3,4) is 10 nm away from the other for its wavelength label, with (1,2) accounting for Fano-shaped dispersions and (3,4) for thin-film dispersions. In (1,2), small wavelength change makes more and more neurons switch their states of activation along the data flow, like the falling dominoes. For the layer next to the output, 25.7% of the neurons switch their states, resulting in an abrupt output change. As a contrast, with only fragmentary neurons switching the states, the outputs of (3,4) have only few changes.

The parameters-to-point forward-mapping NN has advantages on several aspects. First, it has less time-consumption in generating photonic dispersions compared to the available electromagnetic simulation algorithms. For example, it will cost almost 10 min for the RCWA method to simulate only one photonic dispersion of an SOI grating with 200 × 51 pixels (interval wavelength is 3 nm) while just 2.82 s are needed to generate equally sized dispersions for 200 samples by our proposed NN. The second advantage lies in that it can be trained with very few data sets. The inverse-mapping NN usually needs a data set containing 60,000 dispersions (14 GB), still a quite small-volume set in comparison with the library approach. As for our proposed NN case, a 3 GB data set has been already enough. Lastly, the parameters-to-point forward-mapping NN is 200 times smaller than the inverse-mapping one due to its slender architecture, taking up only 1 MB of memory.

### Technology platform

**Fig. 3:**
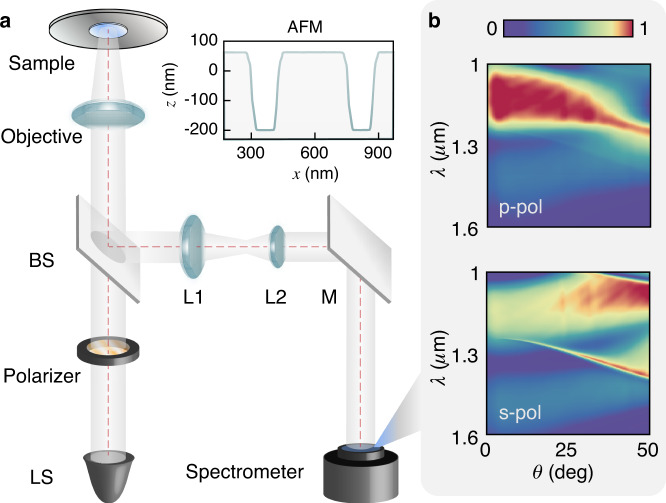
Experimental setup and measured results of an SOI grating. **a** Schematic layout of the home-made ARS. Sample profile of the grating in the top inset. BS beam splitter, L1, L2 lenses, M mirror, LS light source. **b** Dispersions of the sample measured as angle-resolved reflectance (R) spectra

In this section, we introduce the developed measuring methodology to measure the dispersions of SOI gratings. These grating structures are fabricated by using electron-beam lithography on the device layer of an SOI wafer (the thicknesses of the device layer and the buried oxide layer: ~ 270 nm and ~1 μm) with nominally geometric parameters (line widths: 175–300 nm, periodicity: 400–500 nm). The structure areas (400 μm× 400 μm) are much larger than the periodicity, which shield the influence of the grating boundary. The surface topography of an SOI grating measured by AFM is plotted in the top right inset of Fig. 3a.

Taking the angle-resolved reflectance spectra as a holistic characterization of the photonic band dispersion requires both broad spectral imaging and full angular incidences of the incident light. To meet such harsh requirements, we build our ARS based on Fourier analysis, as shown in Fig. 3a. Using Köhler illumination, the sample is illuminated at the front focal plane of the microscope by a halogen lamp. Passing through the incident linear polarizer and focused by an objective lens, the incident beam is convergent and linearly polarized. Then, the reflected beam is Fourier transformed by the same objective lens and imaged to an imaging spectrometer. We can finally observe the dispersions through the two-dimensional charged-coupled-device camera. Here, the objective is 0.95 NA with × 100 magnification, achieving incident angles of up to 50° in near-infrared light. The spot diameter is approximately 100 μm, and measuring range of the spectrometer is from 1 to 1.6 μm. The measured dispersions with p/s-polarized incidence are shown in Fig. 3b, where distinct dispersion bands including sharp Fano-shape features can be clearly observed. The multi-angle detection can be practically realized by the objective lens instead of a mechanical module, enabling us to obtain a dispersion pattern by a single shot^31^.The short measurement procedure and informative dispersions make ARS as a high-through put measuring methodology. In addition, all the optical elements are fixed during the measurement, avoiding additional mechanical noises, which is indispensable since the static light paths offer feasibilities for further calibrations.

### Reconstruction results

To validate our forward-mapping algorithm, we first reconstruct SOI grating profiles from their simulated dispersions. 1,000 p/s-polarized pairs of dispersions with different geometric parameters are calculated by RCWA simulation, making the data set. Following the procedure explained in the subsection ‘Overview of algorithms’, we note that in the parameters-to-point NN, the gradient of parameters can be expressed as $\nabla _{\mathbf{p}}C = \mathop {\sum }\limits_i \nabla _{\mathbf{p}}C_i/m$, where *i* stands for a pixel on the dispersion and *m* for the number of pixels. To test the robustness of our algorithm, reconstructions are performed on both noise-free and noisy data, where noisy data are generated by adding Gaussian noises (*μ* = 0, *σ* = 0.2) to simulate noise-free ones. Statistically, deviations *δ* between ground truths and obtained optimal parameters are shown in Fig. S6. We see that the *δ* for every geometric parameter tends to gather around zero, giving nanometric sensitivity. Results on noise-free and noisy data are similar, validating our algorithm’s robustness. Meanwhile, the 20-s time costs per sample reconstruction makes it commercially available to in-line measurements. Here, deviations in noise-free data are mainly caused by the tiny differences between generated and simulated data. Reconstruction results of other noises are shown in Fig. S7.

**Fig. 4:**
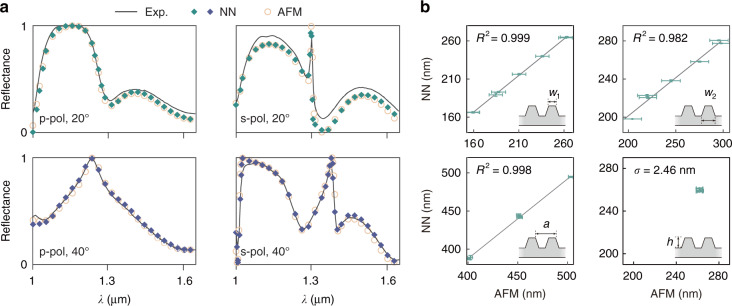
Reconstruction results of experimental data. **a** Detailed comparisons of photonic dispersions: measured by ARS, generated by forward-mapping NN using optimal parameters, and simulated by RCWA using AFM measured parameters. Spectra at two incident angles (20° and 40°) with p/s-polarizations are plotted. **b** Correlations between the results of forward-mapping algorithm (green marks) and AFM measurements (gray line). Error bars are 1 σ

We next turn to the experimental data acquired by ARS. A comparison can be immediately made just after the solving process. One of the reconstruction results is shown in Fig. 4a: Slices of the measured dispersions considered as optimization targets are marked as black lines, in comparison with the corresponding generated dispersions plotted as colored square markers. To verify the generated results, we further use RCWA to simulate the dispersions based on the parameters from AFM measurements, given by yellow ring markers. A good agreement among these three lines tells an excellent performance of our algorithm on the measured data. The complete dispersions and more comparisons of slices are shown in Fig. S9.

Correlations between our forward-mapping NN predicted and AFM measured geometric parameters are shown in Fig. 4b. The reconstruction results of three geometry parameters (the pitch, the top and bottle line widths) for seven SOI gratings are plotted versus the corresponding AFM measurement data, while those of the height are separately shown due to the nearly same height for sample fabrications. NN predictions of the first three parameters achieve a strong linear correlation (*R*^2^ > 0.982) with the AFM measurements, and those of the height are also well reconstructed with mean deviation < 2.46 nm. It should be noted that the AFM measurements are an average within the local regions, since the volume of the probe in AFM is not negligible.

As for the inverse-mapping algorithm, despite its high solving speed (< 1 s) and excellent accuracy (98.7% predicted parameters with the deviation < 1 nm) on simulated data set, the performance on the experimental data is indeed inferior to that of the forward-mapping algorithm (see the Supplementary Information). Given it is impossible to make a data set involving all possible kinds of noises, inverse-mapping NN will always face some unexpected noise. Therefore, experiments are most likely to be mapped to some deviated parameters near the optimal solution, which could not be further finely tuned due to a lack of optimization process. In a word, the inverse-mapping algorithm has its limited advantages. However, if a parameter space is extremely large, forward mapping and inverse mapping may complement mutually as follows: Inverse-mapping NN is able to map the experimental responses to a point in the parameter space near the optimal solution at a high speed, which can be utilized as the initial parameters for the forward-mapping NN to further finely tune to find the optimal solution. At this time, the inverse-mapping NN helps to give a rational starting point instead of a random one, and the forward-mapping NN ensures a robust optimization process in turn.

## Discussion

In this section, we are going to discuss four issues. Firstly, having no contribution to the far field, rapidly attenuated near-field signals are a typical barrier in reconstruction of sub-wavelength structures. Although directly solving for the surface topography without near-field information is hard, alternatively, we can determine grating structures by searching for at solution with a minimum cost function value in parameter space with prior knowledge of the suitable model. As to the parameter space, the more the information obtained in one measurement, the more distinct the difference that appears in the parameter space. A distinct difference in parameter space is helpful for the algorithm to find the minimum cost function, since the large gradient guides the algorithm to reach the unique optimal solution effectively. Due to a large NA objective lens, we can acquire the spectrum information from multiple-angles in one measurement. It is shown that with the wider angle range considered, the topography of the parameter space goes from flat to steep (see the Fig. S11). Besides, with the prior knowledge of the model, potential non-unique solutions are excluded which makes the parameter space have a unique optimal point.

Secondly, we discussed the influences of the noise. Identical noises were generated in the Fano region and the non-Fano region to view the difference in reconstruction results (Fig. S8). It is interesting to compare the perturbation caused by Gaussian blur and bias Gaussian blur. Large parameter deviations only occur when the Fano region is convoluted with a bias Gaussian kernel. For a Gaussian kernel, the convolution only smoothed the peak but did not change the peak position. It shows that Fano-shape dispersion has robustness to the perturbation on the amplitude of the peak. For a bias Gaussian kernel, it led to a shift in peak position, which led to a large variation in the cost function. Note that the location of these peaks is determined by our well-calibrated spectrometer. Thus, the peak position shift should be viewed as a measurement error instead of noises. From another perspective, it shows a high sensitivity of Fano-shape dispersion since only a little parameter deviation will cause a large peak shift.

Thirdly, parameter separation can be visualized by mapping the parameter space.The topography of a pitch-height plane is bowl-like which is easy for the gradient descent algorithm to find the minimum value.The topography of the w_1_–w_2_ space is canyon-like, whose separation is not good as the pitch-height plane. A gradient descent with momentum and a search algorithm were introduced in the algorithm to improve the convergence behavior which yeilded the same result every time with no more than 0.1 nm deviation from different initial parameters. In measurement, changing the azimuth and increasing the acceptance angle may be two feasible schemes to directly change the topography of the w_1_–w_2_ space (Fig. S12).

**Fig. 5:**
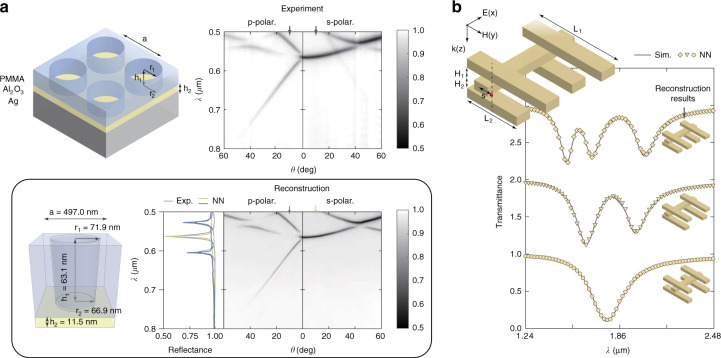
Reconstruction results of 2D grating and 3D plasmon ruler. **a** (top left panel) 2D grating etched in polymethyl methacrylate layer on an aluminum oxide layer and a silver substrate. 2D grating is modeled with five parameters: *a, r*_*1*_*, r*_*2*_*, h*_*1*_ *and h*_*2*_. (top right panel) Measured photonic dispersion patterns of 2D grating with p and s-polarized incident light. (low panel) Corresponding reconstructed parameters of 2D grating. Generated photonic dispersion using forward-mapping NN with reconstructed parameters and detailed slice comparison between experimental and generated dispersion at 10°. **b** 3D plasmon ruler is modeled with five parameters: *L*_*1*_*, L*_*2*_*, s, H*_*1*_*, H*_*2*_. The spectra are shifted upward for clarity. Gray lines are simulated transmittance spectra, which are input into the forward-mapping algorithm for reconstruction. Plasmon-ruler structure diagrams next to the spectra are reconstructed results. Spectra generated by forward-mapping NN are plotted with golden markers

Finally, due to the pixel-by-pixel generation strategy of parameters-to-point architecture, the proposed NN can be flexibly migrated to other models for solving multiple ISPs. To demonstrate its migration ability, we trained forward-mapping NNs on data sets of 2D gratings^41^ and 3D plasmon-ruler structures^42^ for reconstruction, as shown in Fig. 5. In Fig. 5a, we fabricated a 2D grating on the polymethyl methacrylate layer and measured its photonic dispersion patterns with p- and s-polarized incident light. Reconstruction results obtained by using the forward-mapping algorithm are shown in the lower panel of Fig. 5a. In Fig. 5b, we demonstrate the reconstruction of 3D plasmon-ruler structures from simulated transmittance spectra using the forward-mapping algorithm. (Details of 2D grating and 3D plasmon-ruler structure reconstruction are shown in Fig. S14 and Fig. S15.) Since the plasmon-ruler structure has wide potential applications in monitoring macromolecular transformations, the combination of 3D plasmon rulers and the proposed algorithm will pave the road to usage of plasmon rulers in biological and soft-matter systems^42^.

In brief, we have developed a new feasible strategy containing an NN-based algorithm and high-throughput ARS for solving ISPs. It reconstructs SOI gratings with nanometric sensitivity and seconds-level time consumption, covering a wide-range parameter space. Through adopting a parameters-to-point architecture, forward-mapping NN is able to generate photonic dispersions containing sharp Fano-shaped features with high precision, strong robustness, and small volume, which ensures the injectivity and stability for grating reconstructions. It also shows high efficiency when combining the hybrid optimization algorithms, making it available for the industrial in-line data process. The proposed algorithm can also be flexibly migrated to solve ISPs with other models. Furthermore, the ability to acquire the experimental dispersions in a single shot by the Fourier-based ARS is another unique technique for increasing the detecting efficiency. Our strategy has made good predictions against actual noises in accord with the AFM measurements, but with nondestructive nature, which means it could provide a versatile methodology to reconstruct grating profiles as well as other ISPs.

## Materials and methods

### Training and simulations

The training of NN was performed using a single server with a NVidia Tesla V100 graphics card and Intel(R) Xeon(R) Gold 6230 central processing unit. It costs ~ 10 h for generating the data set of a forward-mapping NN and ~5 days for that of an inverse-mapping NN. The training process of forward-mapping NN costs ~ 8 h and that of an inverse-mapping NN costs ~ 6 h.

### AFM measurement

Samples were measured with a commercial AFM (Dimension Icon, Bruker) in tapping mode. The AFM was calibrated using the standard artifacts. A high-aspect-ratio tip probe (TESPA-HAR, Bruker) was used to characterize the trench on the sample. The profile is scanned with 512 pixels/line. For each grating on the sample, the geometrical parameters were analyzed to obtain the average from five different scanned profiles with SPIP software.

## Supplementary information

Supplementary Information for photonic-dispersion neural networks for ISPs.

## Data Availability

The data that support the findings of this study are available from the authors on reasonable request; see author contributions for specific data sets.
