# Correction: Photonic-dispersion neural networks for inverse scattering problems

**DOI:** 10.1038/s41377-021-00635-1

**Published:** 2021-09-15

**Authors:** Tongyu Li, Ang Chen, Lingjie Fan, Minjia Zheng, Jiajun Wang, Guopeng Lu, Maoxiong Zhao, Xinbin Cheng, Wei Li, Xiaohan Liu, Haiwei Yin, Lei Shi, Jian Zi

**Affiliations:** 1 State Key Laboratory of Surface Physics, Key Laboratory of Micro- and Nano-Photonics Structures (Ministry of Education) and Department of Physics, Fudan University, Shanghai, 200433 China; 2 Shanghai Engineering Research Center of Optical Metrology for Nano-fabrication (SERCOM), Shanghai, 200433 China; 3 Institute of Precision Optical Engineering, School of Physics Science and Engineering, Tongji University, Shanghai, 200092 China; 4 National Institute of Metrology, Beijing, 100029 China; 5 Collaborative Innovation Center of Advanced Microstructures, Nanjing University, Nanjing, 210093 China

**Keywords:** Near-infrared spectroscopy, Photonic crystals

Correction to: *Light: Science & Applications*

10.1038/s41377-021-00600-y published online 27 July 2021

After publication of this article^1^, it is noticed this article contained an error. In Results section, the equation ‘${\mathsf{C}} = \vert \Sigma \, \vert {\mathsf{Rd}}{\hbox{-}}\, {\mathsf{Rg}}\vert{2}$’ in the sentence below should be corrected to ‘${\mathsf{C}} = \Sigma \vert {\mathsf{Rd}}{\hbox{-}}\, {\mathsf{Rg}}\vert^{2}$^’^.

The difference between the generated response ${\mathsf{Rg}}$ and the detected response ${\mathsf{Rd}}$ (red block) is described with a cost function, for instance, mean square error ${\mathrm{C}} = \Sigma \vert {\mathsf{Rd}} - {\mathsf{Rg}} \vert {2}$, reflecting in the fluctuations in parameter space.

The original article has been updated.
