# Supplementary material for: Photonic-dispersion neural networks for inverse scattering problems
Source: Light Sci Appl. 2021 Jul 27;10:154. doi: 10.1038/s41377-021-00600-y (PMC8316458; doi:10.1038/s41377-021-00600-y)
Supplement: Supplementary file 1 — Supplementary Information for photonic-dispersion neural networks for ISPs. [file 41377_2021_600_MOESM1_ESM.pdf]

Supplementary Information for photonic-dispersion neural networks for inverse scattering problems

Tongyu Li[1,2,*], Ang Chen[2,*], Lingjie Fan[1,2], Minjia Zheng[1,2], Jiajun Wang[1,2], Guopeng Lu[2], Maoxiong Zhao[1,2], Xinbin Cheng[3], Wei Li[4], Xiaohan Liu[1,5], Haiwei Yin[2], Lei Shi[1,2,5,†], and Jian Zi[1,5,‡]

[1] State Key Laboratory of Surface Physics, Key Laboratory of Micro- and Nano-Photonics Structures (Ministry of Education) and Department of Physics, Fudan University, Shanghai 200433, China

[2] Shanghai Engineering Research Center of Optical Metrology for Nano-fabrication (SERCOM), Shanghai 200433, China

[3] Institute of Precision Optical Engineering, School of Physics Science and Engineering, Tongji University, Shanghai 200092, China

[4] National Institute of Metrology, Beijing 100029, China

[5] Collaborative Innovation Center of Advanced Microstructures, Nanjing University, Nanjing 210093, China

[*] These authors contributed equally to this work.

[†] lshi@fudan.edu.cn

[‡] jzi@fudan.edu.cn

Content

1. Simulation of ARS-measured dispersion patterns

2. Inverse-mapping NN

3. Performance of inverse-mapping NN on actual data.

4. Generation performance of NN with a traditional parameters-to-spectrum architecture

5. Generation performance of NN with a parameters-to-point architecture

6. Details in optimization algorithm

7. Statistical histograms of reconstruction results using forward-mapping algorithm

8. Statistics results of reconstruction from bands with more noise

9. Noise influence on Fano region and non-Fano region

10. Reconstruction results of experimental data

11. Repeatability test of optimization algorithm

12. Parameter space evolution with different acceptance angle

13. Parameter separation

14. Using inverse-mapping NN as initializations

15. Comparison between inverse-mapping and forward-mapping NN

16. Solving inverse scattering problems in 2D grating

17. Solving inverse scattering problems in 3D plasmon-ruler structure

1. Simulation of ARS-measured dispersion patterns

Data set of dispersion patterns measured by Fourier-optics-based angle-resolved imaging spectroscopy (ARS) was generated with rigorous coupled-wave analysis (RCWA) simulation. With a *prior* knowledge, the analyzed grating profile is modeled as isosceles trapezoids using 4 geometric parameters: top line width w_1, bottom line width w_2, pitch a and height h. The grating structures were approximately decomposed into several stairs-like blocks during the simulation. To simulate the full angular incidence of near infrared (NIR) light, we calculated the reflectance for every incident angle and wavelength. When we simulated complete dispersion patterns for inverse-mapping neural network (NN), the incident angle was sampled at 1° intervals within the maximum incident angle provided by the high numerical aperture of 0.95. The wavelength was sampled at 3 nm intervals from 1.0 to 1.65 μm. When we calculated data set for forward-mapping NN with a parameters-to-point architecture, reflectance of points on dispersion patterns with random geometric parameters and coordinates were simulated and stored in the data set labeled with corresponding parameters.

Due to the C_2 symmetry of the grating model, only the positive incident angle was simulated. Additionally, the total power received by ARS at each angle θ contains the contribution from both specular reflection and high-order diffractions. Using Laue equation to track all of light contribution, the total received power can be written as

$$\mathrm{I(k)} = \sum_{n=\lceil -|k_{\parallel,m}-k_{\parallel}|/|G| \rceil}^{\lfloor |k_{\parallel,m}+k_{\parallel}|/|G| \rfloor} \mathrm{I_0}(k_{\parallel} + nG)\mathrm{R}_{-n}(k_{\parallel} + nG) \qquad \text{(S1)}$$

where $\lceil \dots \rceil$ and $\lfloor \dots \rfloor$ represent ceil and floor operator, k is the wave vector, subscript $\parallel$ stands for the projection of the wave vector onto the grating plane, $\mathrm{G} = 2\pi\hat{a}/a$ is the reciprocal lattice vector of the grating, $\mathrm{k_m}$ is the wave vector incident at the maximum incident angle, $\mathrm{I_0(k)}$is the intensity of incident light, and $\mathrm{R_n}$ is the reflectance of the *n*th-order diffracted light. The reflectance defined as $\mathrm{I(k)/I_0(k)}$ is calculated for every angle and wavelength to form the simulation of ARS measurement. These simulation results of different grating parameters are finally collected in a data set labeled with their geometric parameters.

2. Inverse-mapping NN

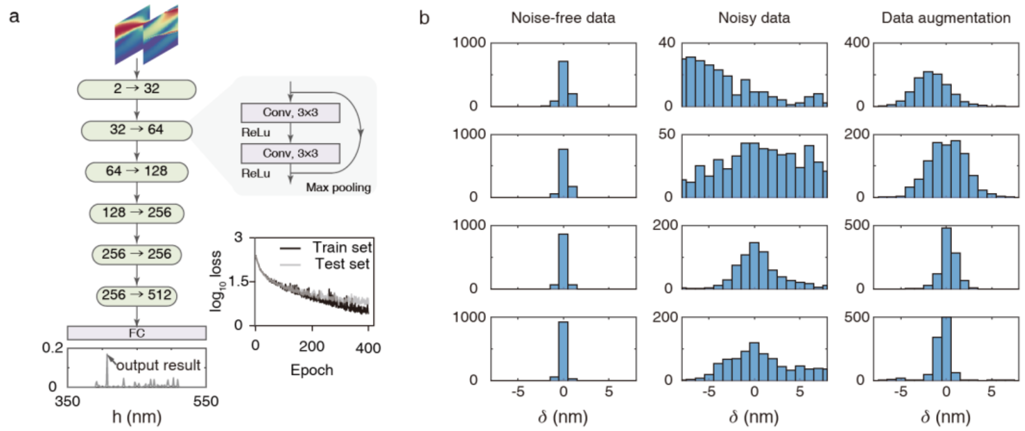

Figure S1: **Inverse mapping NN a,** Architecture of inverse mapping NN. The neural network contains 6 residual blocks and 2 fully connected layers, where each residual block consists of 2 convolutional layers, an in-to-out shortcut connection and a max pooling layer. Training loss is in the down inset. **b,** Statistical histograms of reconstruction result by using inverse mapping NN. Left column is the deviation of reconstruction result on noise-free test set, middle column is the performance on noisy test set (Gaussian noises μ=0, σ=0.1), and right column is the performance of NN after date set augmentation on the same noisy test set.

A deep convolutional neural network with residual learning technique is trained to learn the inverse mapping from data space to model space for reconstructing the grating profiles, whose architecture is shown in Fig.S1. The neural network contains 6 residual blocks and 2 fully connected layers, where each residual block consists of 2 convolutional layers and an in-to-out shortcut connection. Here, shortcut connections skip the convolutional layers to simply perform identity mapping whose outputs are directly added to the outputs of the stacked convolutional layers. After the output of the convolutional layers, the subsequent fully connected layers play roles to map those extracted feature maps into probability distribution of each geometric parameter as the final outputs. The neural network is trained on the dataset by minimizing the cost function that is defined by Eq. S2,

$$C(g, p; R_{in}, \theta) = -\frac{1}{m}\sum_{i=1}^{m}\sum_{j=1}^{n}\sum_{k=1}^{q}\left\{\delta_{g(i,k),r(j,k)} \ln \frac{\exp[p_\theta(z_{ijk}|R_{in})]}{\sum_{l=1}^{n}\exp[p_\theta(z_{ilk}|R_{in})]}\right\} \tag{S2}$$

where the cross entropy function is used to characterize distance between the predicted probability distribution *p* of each geometric parameter and the ground true distribution $\delta(x-g)$ and then averaged over all the training data set; R_{in} is the input of network, *z* is the output of neural network, *m* is the size of data set, *n* is the quantity of output, and *q* is the number of feature parameters in the model. The coordinate of each probability distribution is not infinite, instead, a prior range, which defaults the probability distribution outside the range is zero. Then, the predicted probability distribution is discretized into *q* points with 1 nm as the interval. Each element of the matrix **g** and **r** stands for the label of training data set and the discrete coordinate of the output respectively.

The objective of the optimization is to narrow the difference between the prediction and the ground true by iteratively tuning parameters *θ* of the neural network, which can be described as

$$\theta = \arg\min_{\theta'} C(g, p; R_{in}, \theta) + \alpha\|w\|_2 \quad \text{(S3)}$$

where parameters θ, including the kernels, weights and biases, are initialized randomly with normal distribution and optimized for 400 epochs by Adam optimizer with batch size 1024. Data set contains 60, 000 pairs of dispersion patterns for p- and s- polarized incident light. The initial learning rate is 0.001 and decay 10 times every 100 epochs. Some training tracks such as dropout and l_2 regularization are added to FC layers during training time to prevent overfitting with keep probability 0.8 and α=0.001 respectively. Batch normalization is introduced after each convolution layer and before activation to reduce the influence of learning rate settings and parameters initialization on training.

Inverse-mapping NN had a novel performance on noise-free test set with 1, 000 pairs of dispersion patterns for p- and s- polarized incident light, as shown in the first column of Fig.S1. While, when we verified its performance on a test set with Gaussian noise (μ=0, σ=0.1), predicting results had large deviations from labels, as shown in the middle column. To improve robustness of NN, we further trained inverse-mapping NN again on augmented data set by adding random Gaussian noise to these photonic dispersion patterns. After training, NN's performance on the same noisy test set improved distinctly, as shown in the right column. It means that inverse-mapping NN cannot give a precise prediction from a dispersion pattern with unexpected noise, but its robustness can be enhanced by data set augmentation with corresponding type of noise. To achieve robust prediction from measured photonic dispersion with actual noise, we introduced several potential types of random noise to simulate actual measurement, including Gaussian noise, low-frequency noise and Gaussian blur. These types of noise were generated and added to dispersion bands during the training process.

3. Performance of inverse-mapping NN on actual data

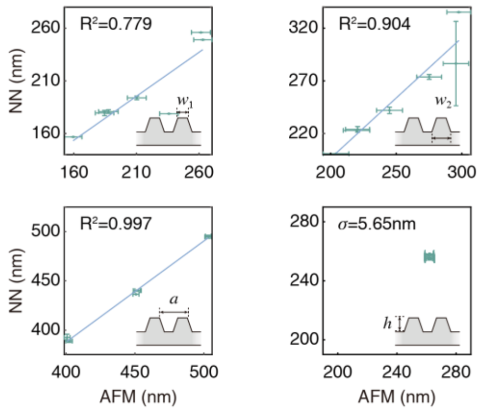

Figure S2: **Performance of inverse-mapping NN on actual data.** To achieve robust prediction from measured photonic dispersion with actual noise, we introduced several potential types of random noise to simulate actual measurement, including Gaussian noise, low-frequency noise and Gaussian blur. These types of noise were generated and added to dispersion bands during the training process. After data set augmentation, inverse-mapping NN is performed on actual data (the data correspond to Fig. 5 of the manuscript). Since these introduced noises cannot cover all experimental noises, for some samples, some predictions of NN having large deviations from the AFM data.

4. Generation performance of NN with a traditional parameters-to-spectrum architecture

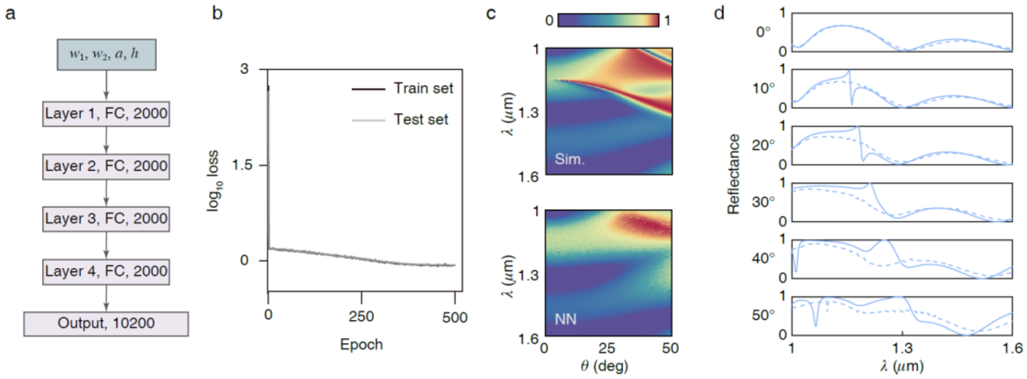

Figure S3: **Generation performance of NN with a traditional parameters-to-spectrum architecture. a,** NN had 4 hidden layers with 2, 000 neurons per layer. Geometric parameters of SOI grating were input to NN, and the output was an array of reflectance which were further reshaped into a 2D dispersion pattern with 200×51 pixels. NN was trained on a data set containing 60, 000 dispersion patterns (about 14 GB) for s-polarized incident light and tested on another data set whose examples never participated in the training process. Mean square error (MSE) was used as the cost function to characterize the difference between the generated patterns and ground truths. The weight parameters in NN were trained using an Adam optimizer with batch size 1024 for 500 epochs. The initial learning rate was set to be 0.005, and was lowered by 10 times every 100 epochs. **b,** Training loss. **c and d,** Comparison between simulated and generated photonic dispersions. Here, around 6×10^7 parameters were used to construct the NN, but its generation performance was unappealing. It could be found that NN was able to generate these thin-film-like features but fail to Fano-shaped features. We believed that with more complex NN architecture and more NN parameters used, the performance of the NN with a parameters-to-spectrum architecture would improve. While, at this time, enormous parameters will make the NN too cumbersome to use.

5. Generation performance of NN with a parameters-to-point architecture

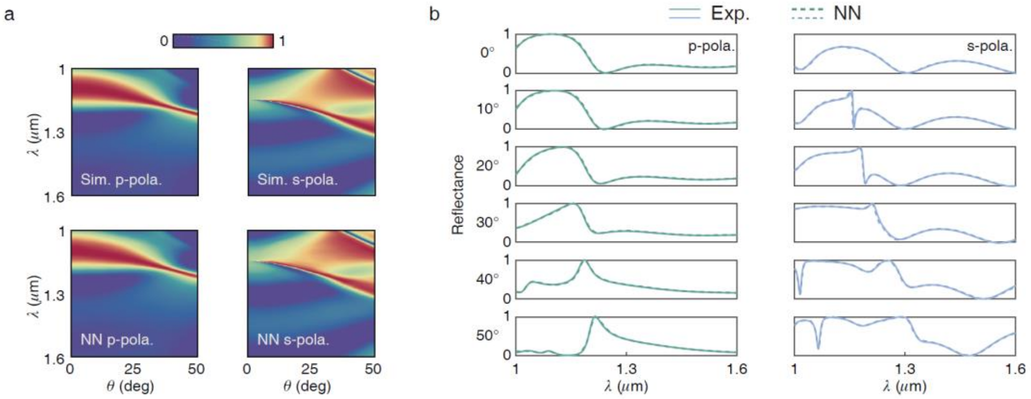

Figure S4: **Generation performance of NN with a parameters-to-point architecture.** The data correspond to Fig. 2 c-d of the manuscript. **a,** Generation performance of photonic dispersion with p/s polarized light. **b,** Detailed comparison between the slices of generated and simulated dispersion patterns.

6. Details in optimization algorithm

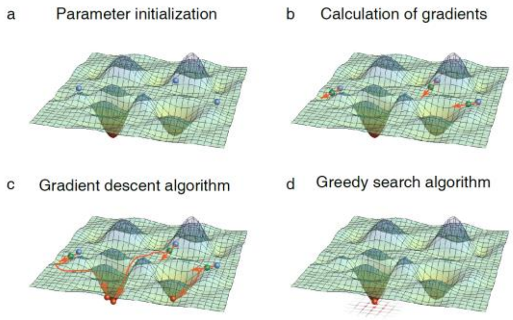

Figure S5: **Visualization of hybrid optimization algorithm.** The proposed hybrid optimization algorithm contains gradient descent algorithm and greedy/search algorithm. Minimizing a cost function is the objective of this algorithm. Here, cost functions have many options and we choose MSE for reconstruction task. **a,** Several initial points (10 for example) are chosen randomly on parameter space as the beginning. The corresponding dispersion patterns of these points are subsequently generated by forward-mapping NN. Then, cost function between generated and detected dispersion is obtained. By using back propagation algorithm, we calculated the gradients of parameters for gradient descent algorithm. After iterating step **b** and **c** 70 times， the iteration was stopped prematurely. The point with the minimum cost function value is chosen as the candidate solution. **d,** Greedy/search algorithm starts from the candidate solution to find the final solution.

Algorithm 1 Our proposed algorithm for optimizing n grating parameters ties in with the trained forward-NN. To find the optimal solution, the optimization strategy is generally divided into two stages: 1. Using gradient descent to optimize parameters starting from m sets of initial values θ_0. $m \times n$ parameter matrix θ_0 is initialized as uniform distribution within a *prior* range; 2. Greedy search begins from the candidate solution to scan the parameter space for the final reasult. Algorithm alternately searches the optimal solution on the Pitch-Height space and W_1-W_2 space to obtain the final result.

Require: R_m: A measured photonic dispersion pattern;
Require: $C(R_m, R_{NN}(\theta))$: Cost function with parameters θ;
Require: θ_0: Initial parameter matrix;
Require: α: Stepsize; (Suggested default: 0.001)
Require: $\beta \in [0, 1)$: Exponential decay rates for stepsize; (Suggested default: 0.99)
Require: δ: Search scope;

$t \leftarrow 0$ (Initialize timestep)
$v \leftarrow 0$ (Initialize velocity)
while θ_t not converged **do**
  $t \leftarrow t + 1$
  $R_{NN} \leftarrow R_{NN}(\theta_{t-1})$ (Compute n sets of dispersion patterns with NN)
  $g_t \leftarrow \nabla_\theta C_t(R_m, R_{NN})$ (Get gradients at timestep t through BP)
  $v_t \leftarrow \epsilon v - \alpha g$ (Update velocity)
  $\theta_t \leftarrow \theta_{t-1} + \beta v_t$ (Update parameters)
end while
$\theta_{c,0} \leftarrow \theta_t[\text{where}(C_t == \min(C_t)), :]$ (Choose candidate parameters with minimum C_t)
$t \leftarrow 0$ (Initialize timestep)
while $\theta_{c,t}$ not converged **do**
  $t \leftarrow t + 1$
  $\Delta\Theta_i \leftarrow \text{linspace}(\theta_{c,t-1}[i] - \delta_i, \theta_{c,t-1}[i] + \delta_i, N_i)(i = 1, 2)$
  $\Theta_{a,h} \leftarrow \Delta\Theta_1 \bigotimes \Delta\Theta_2 \bigotimes \theta_{c,t}[3 :]$ (Construct a a-h space at t)
  $C_{c,t} \leftarrow C_{c,t}(R_m, R_{NN}(\Theta_{a,h}))$(Compute cost function in a-h space)
  $\theta_{c,t-0.5} \leftarrow \Theta_{a,h}[\text{where} C_{c,t} == \min(C_{c,t}t)]$(Update candidate parameters)
  $\Delta\Theta_i \leftarrow \text{linspace}(\theta_{c,t-0.5}[i] - \delta_i, \theta_{c,t-0.5}[i] + \delta_i, N_i)(i = 3, 4)$
  $\Theta_{w1,w2} \leftarrow \theta_{c,t-0.5}[: 2] \bigotimes \Delta\Theta_3 \bigotimes \Delta\Theta_4$(Construct a w_1-w_2 space at $t + 0.5$)
  $C_{c,t+0.5} \leftarrow C_{c,t+0.5}(R_m, R_{NN}(\Theta_{w1,w2}))$(Compute cost function in w_1-w_2 s-pace)
  $\theta_{c,t} \leftarrow \Theta_{w1,w2}[\text{where}(C_{c,t+0.5} == \min(C_{c,t+0.5}))]$(Update candidate parameters)
end while
return $\theta_{c,t}$: reconstructed grating parameters;

Table S1: **Pseudocode of optimization process.** In the first step, gradient descent algorithm starts from 10 initial points to approach the global minimum value with the step length α =0.001 and exponential decay coefficient β =0.99. After convergence, parameters with the minimum cost function was selected as the candidate solution. In the second step, greedy search is then started from the candidate solution. We scanned pitch-height space and w1-w2 space alternately to get reconstruction results. Due to the searching range in our algorithm is an expanded space of radius 5 nm centered at the candidate point, search process is much efficient and always converges in 2~3 iterations.

7. Statistical histograms of reconstruction results using forward-mapping algorithm.

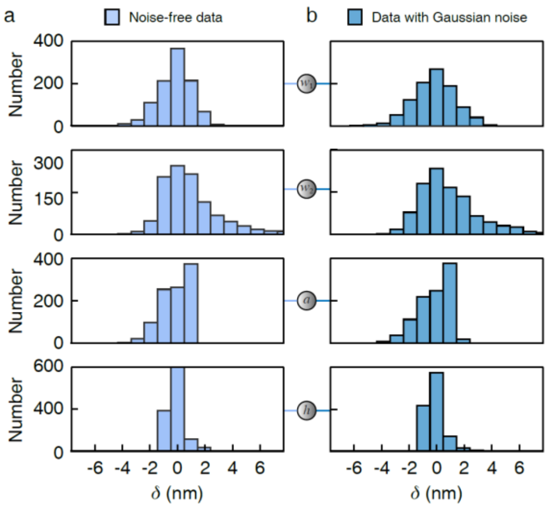

Fig. S6 Statistical histograms of reconstruction results using forward-mapping algorithm. a, Deviations on noise-free dispersions. **b** Same as **a** but with Gaussian noises (μ=0, σ=0.2).

8. Statistics results of reconstruction from bands with more noise

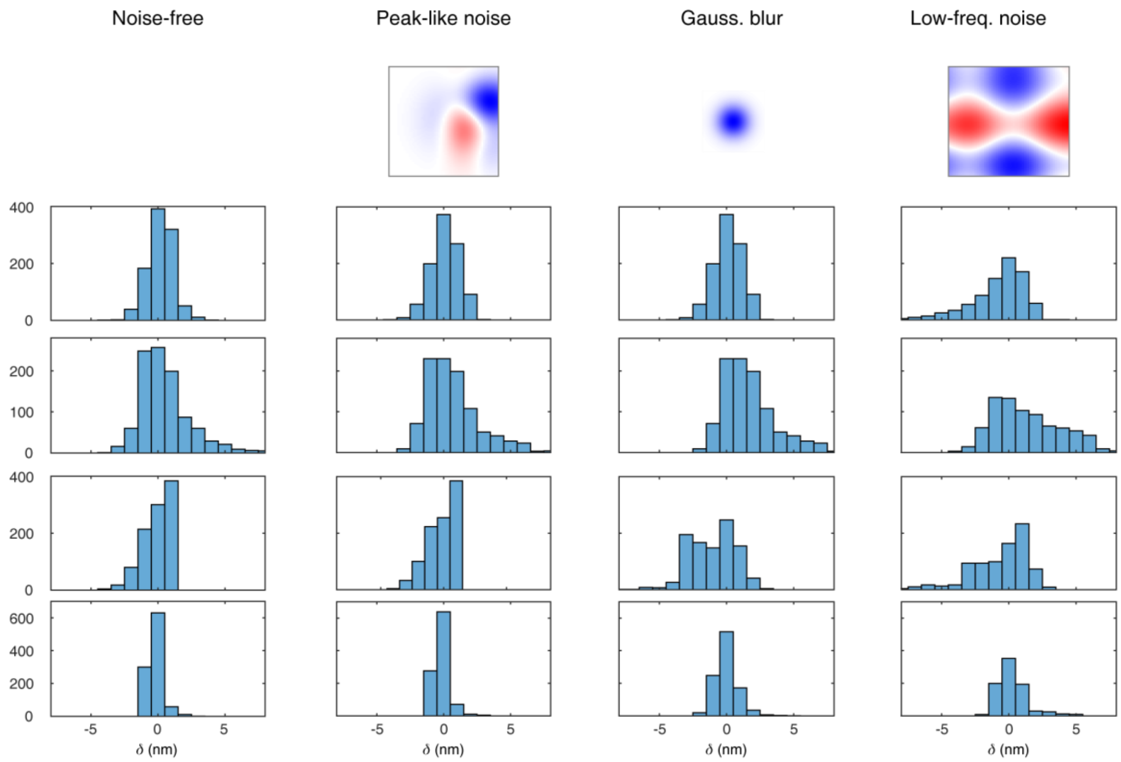

Figure S7: **Statistics results of reconstruction from bands with more noise.** Statistics results of reconstruction from bands with more noise (peak-like noise, Gaussian blur and low-frequency noise). Peak-like noise is formed by generating a random number (2~5) of Gaussian peak ($A\in$[-0.1, 0.1], $\sigma\in$[10, 30] pixels) at random location on the simulated bands. Gaussian blur is performed by convolution with 5×5 pixels Gaussian kernel. Low-frequency noise is a kind of integral perturbation which is composed of a random number (2~5) of horizontal and vertical sine-like noise.

9. Noise influence on Fano region and non-Fano region

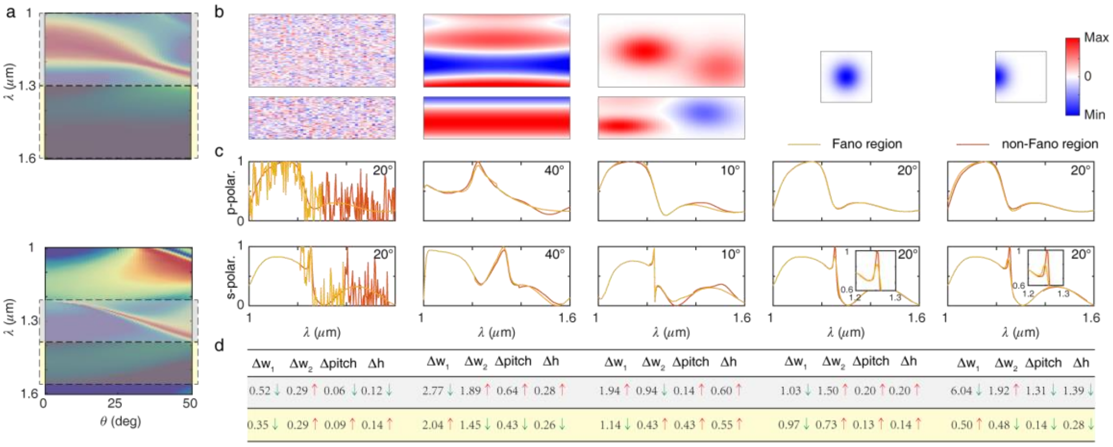

Figure S8: **Noise influence on Fano region and non-Fano region. a**, Photonic band with s- and p- polarization and are divided into Fano region (gray block) and non-Fano region (yellow block). Identical noises were generated in Fano region and non-Fano region respectively to view the difference in reconstruction result. **b**, 5 types of noises are considered: white noise, low-frequency noise, peak-like noise, Gaussian blur and bias Gaussian blur. **c**, Slices of photonic bands. **d**, Reconstruction results. It is interesting to compare the influence caused by Gaussian blur and bias Gaussian blur. Large parameters' deviations only occur when Fano region is convoluted with a bias Gaussian kernel. For a Gaussian kernel, the convolution only smoothed the peak but didn't change the peak position. It shows that Fano-shape dispersion has robustness to the perturbation on the amplitude of the peak. For a bias Gaussian kernel, it led to a shift in peak position at the same time which led large variation in MSE. Note that the location of these peaks is determined by our well-calibrated spectrometer. Thus, peak position shift should be viewed as a measurement error but not noises.

10. Reconstruction results of experimental data

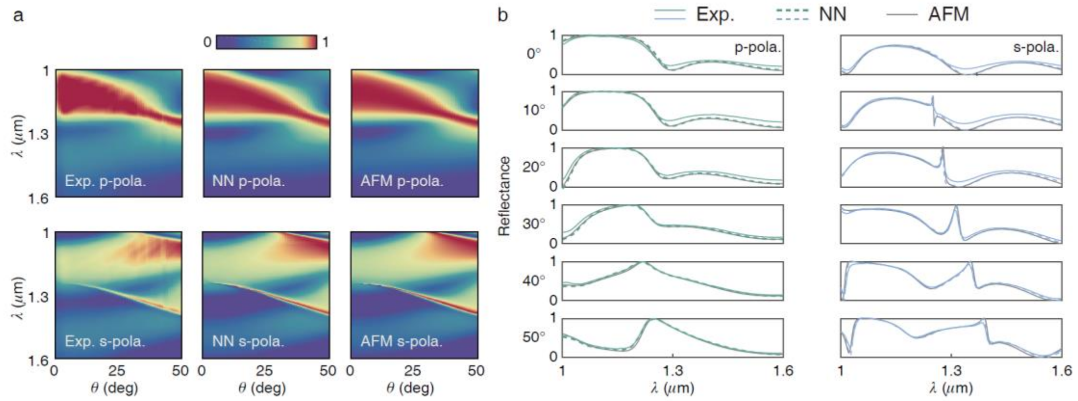

Figure S9: **Reconstruction results of experimental data.** The data correspond to Fig. 5 of the manuscript. **a**, Comparisons of photonic dispersions: measured by ARS, generated by forward-mapping NN using optimal parameters, and simulated by RCWA using AFM measured parameters. **b**, Further comparisons of slices.

11. Repeatability test of optimization algorithm

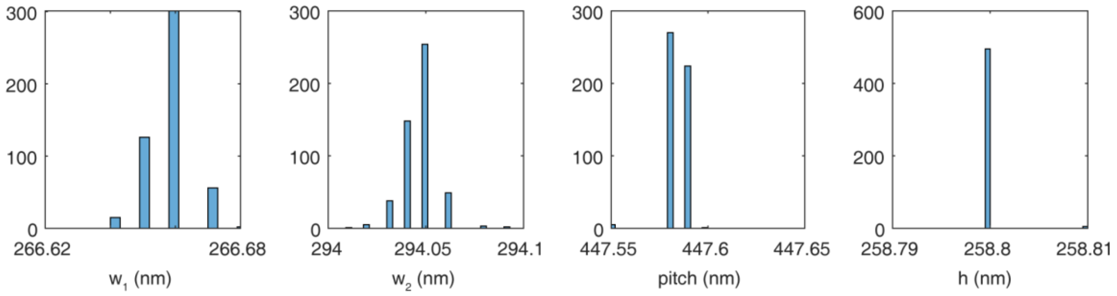

Figure S10: **Repeatability test of optimization algorithm.** Statistical result of reconstruction from measured photonic band illustrated in Fig. 5(a) with 500 random initial value. The algorithm can find the same solution every time with no more than 0.1 nm deviation.

12. Parameter space evolution with different acceptance angle

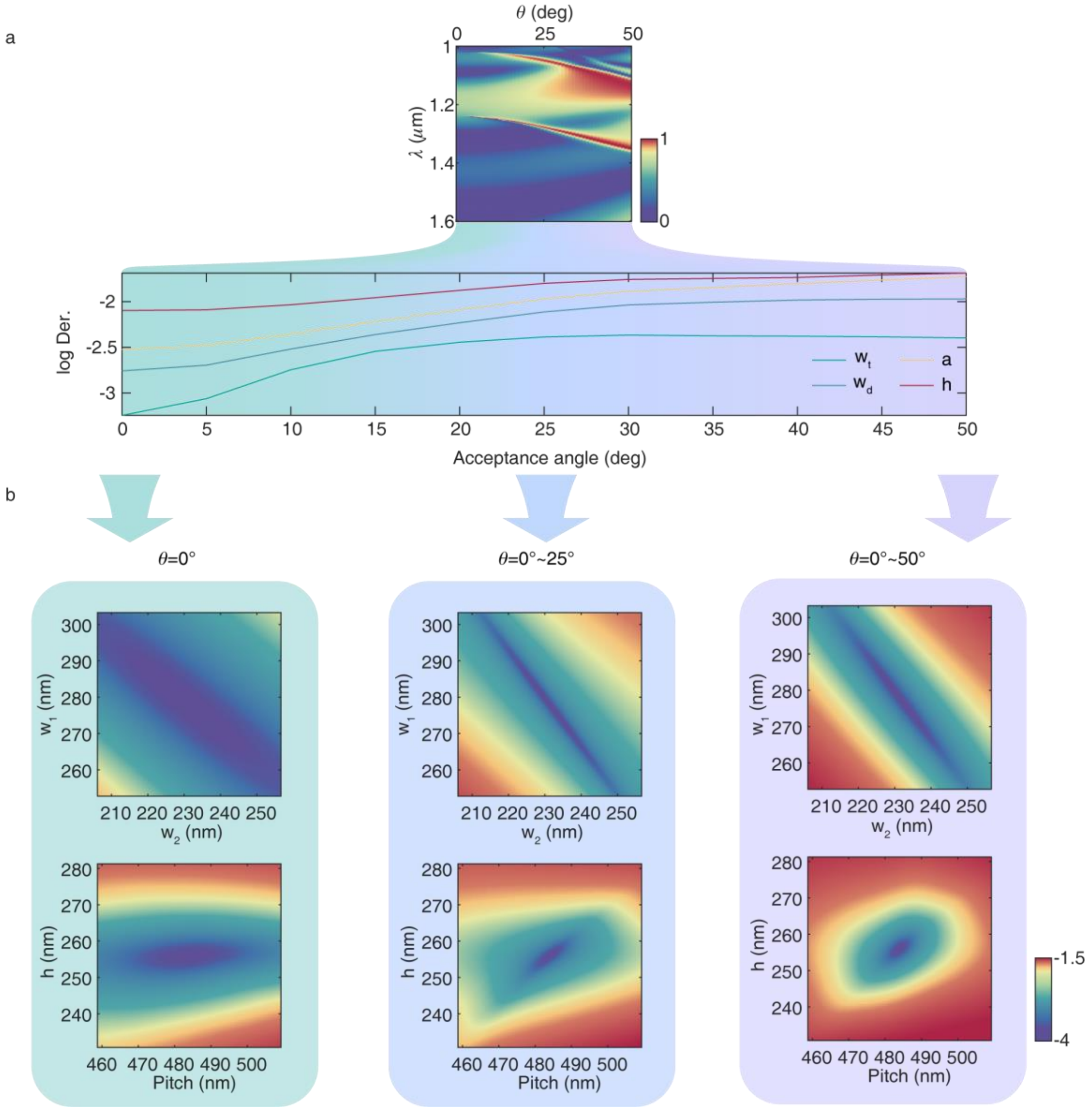

Figure S11: **Parameter space evolution with different acceptance angle a**, horizontal axis stands for the acceptance angle range, and vertical axis for log MSE between two simulated photonic bands with 10 nm variation in geometrical parameters. **b**, parameter space visualization with different acceptance angle range, z-axis of parameter space is log MSE. With wider angle range considered, the difference between two photonic band increased significantly. The minimal variation of parameter is 0.5 nm between two neighboring points.

13. Parameter separation

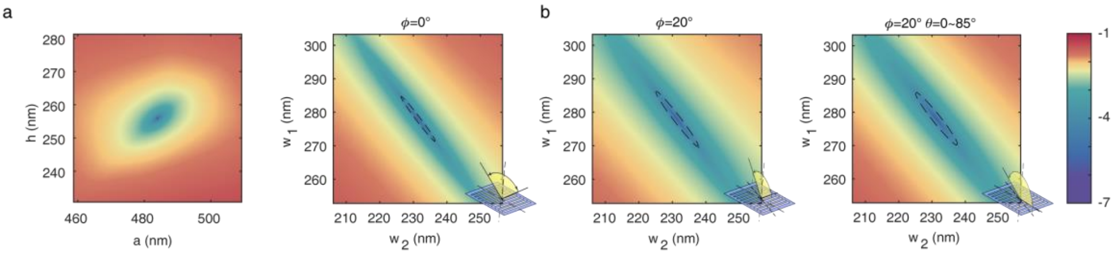

Figure S12: **Parameter separation. a**, MSE distribution on pitch- height space and w_1-w_2 space. **b**, by changing azimuth from 0° to 20° and increasing acceptance angle to 85°, the narrow axis of the canyon is gradually widened that improves the convergence behavior of algorithm from every direction.

14. Using inverse-mapping NN as initializations

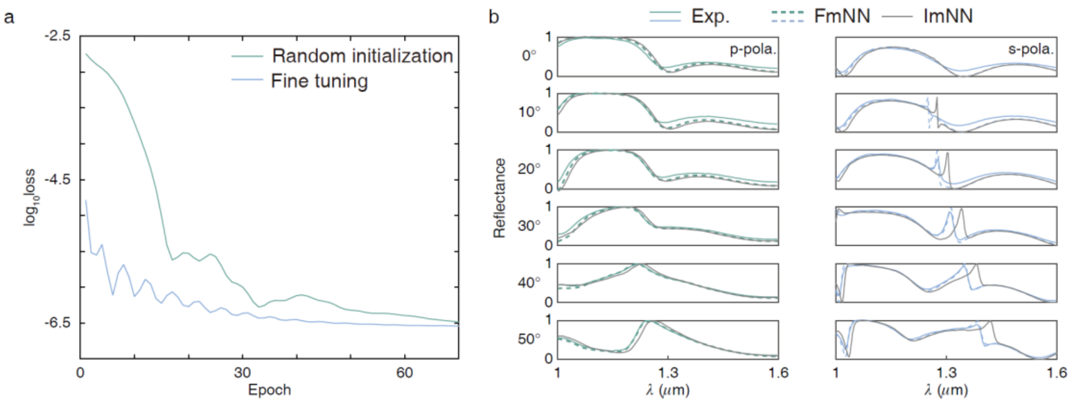

Figure S13: **Using inverse-mapping NN as initializations. a** shows the evolution of the cost function with different initialization. Predictions of inverse-mapping NN correspond to Sec. 3 of the Supplementary Information. Initialization of the green line is randomly generated, while that of blue line is the prediction of inverse-mapping NN. Despite of some deviations, the output of inverse-mapping NN can be viewed as a good initialization indicating an approximate location of optimal solution. On the other side, forward-mapping algorithm finely tune the biased result of inverse-mapping NN (w_1, w_2, a, h)=(252.0, 327.0, 452.0, 258.0) nm to the optimal solution (269.0, 299.6, 441.5, 259.5) nm. **b**, Detailed comparison of photonic dispersion slices before and after fine tuning. FmNN stands for forward-mapping NN, ImNN for inverse-mapping NN. Gray lines are simulated dispersion slices using the prediction results of inverse-mapping NN. Green lines are generated dispersion slices after fine tuning.

15. Comparison between inverse-mapping and forward-mapping NN

	Data set scale	Algorithm scale	Time consumption	Robustness	Real-time comparison
Inverse-mapping	65, 000 patterns (30 GB)	400 MB	<1 s	×	×
Forward-mapping	3 GB	1 MB	~20 s	√	√

Table S2: **Comparison between inverse-mapping and forward-mapping NN.**

16. Solving inverse scattering problems in 2D grating

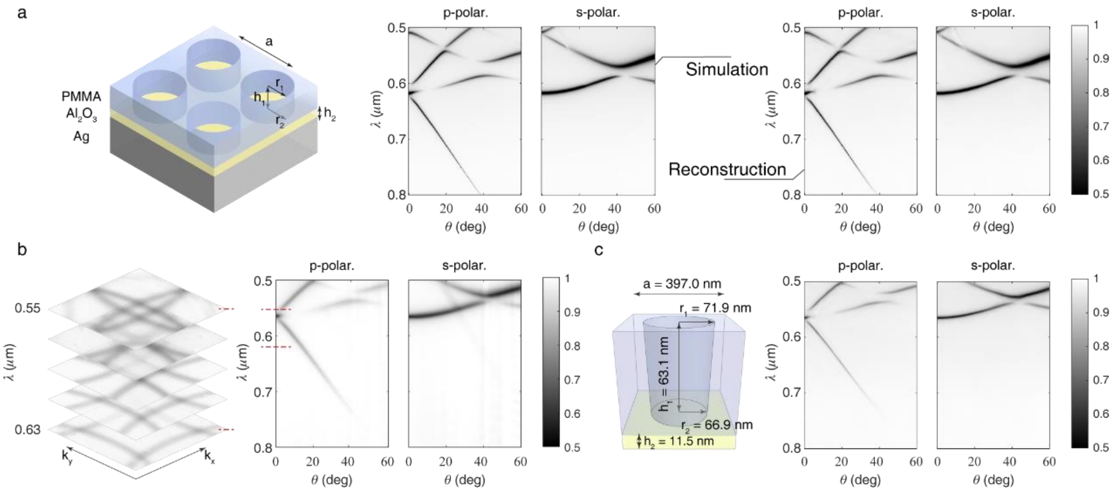

Figure S14: **Solving inverse scattering problems in 2D grating.** **a**, 2D grating reconstruction can be a possible generalization direction of our method. 2D grating etched in PMMA layer on an aluminium oxide layer and an Ag substrate was considered. Comparison of simulated photonic band and generated photonic dispersion using reconstruction parameters are shown on the right side. Ground truths of 5 parameters are r1 = 100.0 nm, r2 = 95.0 nm, a = 435.0 nm, h1 = 75.0 nm and h2 = 18.0 nm, and the reconstruction results are r1 = 99.4 nm, r2 = 95.7 nm, a = 435.6 nm, h1 = 75.0 nm and h2 = 18.0 nm. **b**, Measured photonic bands and iso-frequency contours. Photonic bands were input into algorithm to reconstruct grating's profile, and iso-frequency contours reflected symmetry of 2D grating. **c**, Reconstruction results of 2D grating.

17. Solving inverse scattering problems in 3D plasmon-ruler structure

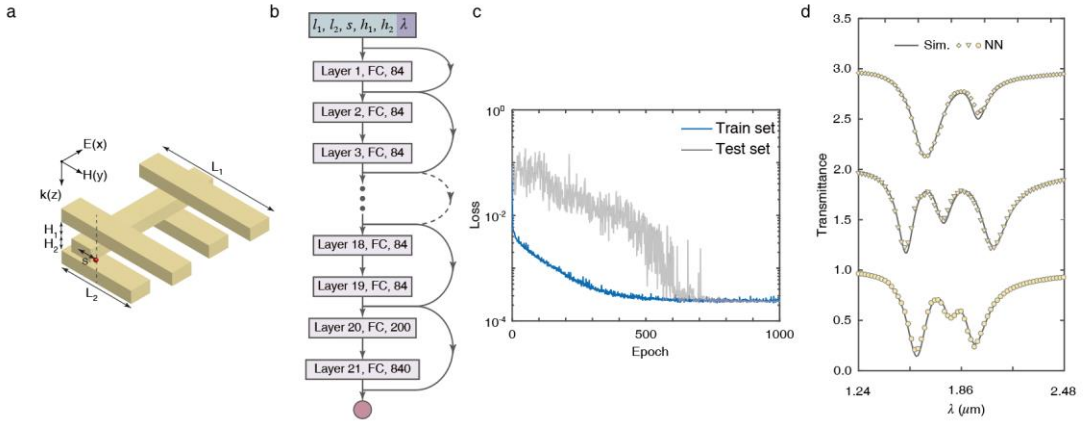

Figure S15: **Solving inverse scattering problems in 3D plasmon-ruler structure. a**, Schematic diagram of 3D plasmon-ruler structure. 3D plasmon ruler is modeled with 5 parameters: L_1, L_2, s, H_1, H_2. **b**, Forward mapping NN architecture. Data set of transmittance spectra are simulated by finite-different time-domain method. 3.5×10^7 transmittances with different geometric parameters and wavelength are calculated for training. Data set was calculated with two servers with Intel(R) Xeon(R) Gold 6230 central processing units for 2 weeks. The training process was performed on a NVidia Tesla V100 graphics card. The initial learning rate is 0.001 and decay 10 times every 100 epochs. **c**, Training loss. **d**, More reconstruction results. Spectra are shifted upward for clarity. Wavelength interval is 4.96 nm. Ground truths (L_1, L_2, s, H_1, H_2) of shown structures (from bottom to top) are (330.0, 300.0, 35.0, 30.0, 20.0) nm, (330.0, 280.0, 45.0, 20.0, 20.0) nm and (350.0, 290.0, 10.0, 30.0, 40.0) nm, and corresponding reconstruction results are (330.5, 300.1, 34.6, 31.2, 19.3) nm, (328.7, 279.6, 44.4, 20.3, 19.7) nm and (351.1, 289.2, 9.8, 31.2, 38.9) nm. Ground truths (L_1, L_2, s, H_1, H_2) of structures in Fig. 6b are (340.0, 340.0, 0.0, 30.0, 30.0) nm, (340.0, 330.0, 40.0, 30.0, 30.0) nm and (340.0, 270.0, 40.0, 30.0, 30.0) nm which are as same as that of Fig. 2 in Ref.[42]. Corresponding reconstruction results are (340.4, 339.6, 0.0, 29.4, 30.2) nm, (340.5, 330.4, 38.8, 29.2, 29.7) nm and (339.8, 270.6, 39.6, 30.5, 29.3) nm
